# What makes work sustainable for adults with ADHD? Interpreting workplace strain and support through Reddit data

**DOI:** 10.3389/fpsyg.2026.1820248

**Published:** 2026-06-15

**Authors:** Tiffany Payton Jameson, Mirit K. Grabarski, Martin Morales Arana, Paul Angelo Santos

**Affiliations:** 1 Grit & Flow, Laguna Hills, CA, United States; 2 Faculty of Business Administration, Lakehead University, Thunder Bay, ON, Canada

**Keywords:** ADHD, ADHD at work, job demands-resource model, neurodiversity, organizational design, psychological safety, sustainable work systems, workplace inclusion

## Abstract

Attention-Deficit/Hyperactivity Disorder (ADHD) is increasingly recognized within organizational research, yet workplace scholars often examine isolated challenges or strengths rather than situating these experiences within perceived patterns of work design and support. Drawing on the Job Demands–Resources (JD–R) framework as a sensitizing lens, this qualitative study examines how contributors to a large ADHD-focused online community describe workplace strain and support within online community discourse. We analyzed 277 workplace-related discussion threads from the Reddit forum r/ADHD using reflexive thematic analysis. Across accounts, workplace features such as meetings, deadlines, and disclosure were not described as either inherently demanding or supportive. Instead, contributors emphasized that these conditions were experienced differently across contexts, particularly regarding autonomy, performance management, structuring of work, and identity-related dynamics. The findings suggest that workplace strain and sustainable functioning were patterned around concepts such as the social impact of disclosure, work design aspects and invisible labor. Importantly, these results reflect collective meaning-making within a large online ADHD community rather than direct representations of organizational structures or causal mechanisms. The study provides a contextualized, discourse-informed interpretation of JD–R processes in ADHD-related workplace narratives and offers hypothesis-generating insights for future empirical research.

## 1 Introduction

Attention-Deficit/Hyperactivity Disorder (ADHD) is a neurodevelopmental condition characterized by persistent inattention and/or hyperactivity–impulsivity, affecting approximately 2.5%–3% of adults worldwide (Faraone et al., 2021; Song et al., 2021). Long framed as a childhood disorder associated with disruptive classroom behavior, ADHD has been stigmatized and stereotyped, often obscuring the cognitive strengths of those diagnosed. Despite comparable or superior intellectual ability, adults with ADHD frequently experience occupational difficulties (Jangmo et al., 2021), suggesting that work outcomes may reflect misalignment between job requirements and available support rather than deficits in capability. Recent research on employment among adults with ADHD has expanded alongside the rise of the neurodiversity paradigm, which conceptualizes neurological differences as natural cognitive variation rather than inherent deficits (Chapman, 2020; Doyle, 2020). Increased workplace neurodiversity initiatives and rising adult diagnoses, particularly among historically underrecognized groups, have further amplified organizational interest in ADHD (Faraone et al., 2021).

Empirical research identifies several workplace challenges associated with ADHD. Among previously identified challenges are executive functioning difficulties, including organizational skills, sustained attention, working memory, and cognitive flexibility (Hotte-Meunier et al., 2024; Liebel et al., 2023), which are foundational for effective job performance (Chan et al., 2021), particularly in terms of task management, time management, and interpersonal dynamics (Liebel et al., 2023). Physical and mental health factors, including heightened emotional reactivity and experiences described as rejection sensitivity dysphoria, also contribute to occupational strain (Faraone et al., 2021; Liebel et al., 2023; McDowall et al., 2025). Turjeman-Levi et al. (2024) further linked executive functioning difficulties to elevated burnout. Medication management introduces additional variability in daily functioning (Cortese et al., 2018; Faraone et al., 2021).

Concurrently, strengths associated with ADHD may support occupational success. Reported strengths include creativity, divergent and innovative thinking, hyperfocus, multitasking, complex problem-solving, mentoring others, and empathy (Hoogman et al., 2020; Liebel et al., 2023; McDowall et al., 2025; Schippers et al., 2022). Core traits such as hyperfocus and hyperactivity/impulsivity have demonstrated both beneficial and detrimental effects depending on contextual conditions (Wiklund et al., 2016). Daily-level evidence supports this variability. Weinhardt et al. (2025) found that symptom expression fluctuated with structural resources, task characteristics, and work engagement. Similarly, Crook and McDowall (2024) identified that the interaction between individual traits and environmental supports shapes both authentic and hard-won career success. Recent qualitative research adopting a sustainable career ecosystem perspective further demonstrates that ADHD differentially impacts sustainability indicators, particularly productivity, and that local ecosystem actors (e.g., family, colleagues, therapists) significantly shape long-term career trajectories (Grabarski et al., 2025; Lauder et al., 2022).

At the intersection of challenges and strengths, adaptive strategies emerge. Identified strategies include medication use, self-awareness, externalizing tasks, clear communication, task switching, managing distractions, modifying meeting structures, adapting work modality (remote, hybrid, or in-office), and leveraging collegial support (Grabarski et al., 2025; Liebel et al., 2023). Many of these strategies function as structural, social, or personal resources. Therefore, the current body of research suggests that occupational outcomes for adults with ADHD are dynamic and contingent upon the interaction between ADHD-related regulatory variability and the demands and resources present in the work environment. We argue that this interplay aligns with the Job Demands-Resources (JD-R) model (Demerouti et al., 2001) as a theoretically robust framework for understanding burnout, engagement, and employment sustainability among adults with or who identify with ADHD.

The Job Demands-Resources (JD-R) theory (Demerouti et al., 2001) explains how different aspects of the workplace can lead to stress and burnout, and how these aspects can be managed to achieve more positive outcomes. The theory describes work settings in terms of demands (aspects that require physical and/or psychological effort, for example, physical tasks, interacting with customers, etc.) and resources (aspects that support performance, foster growth, and relieve pressure from demands). High demands are likely to lead to strain and, eventually, burnout, while resources are likely to increase engagement (Bakker and Demerouti, 2017).

The theory has been extensively used to explain how demands and resources interact not only in the context of well-being (minimizing stress and burnout) but also to predict work performance, which is linked to engagement (Hakanen et al., 2005). Empirical studies have found that JD-R processes fluctuate within-person across days, and that enacting strategies such as job crafting to seek additional resources can improve performance, whereas job crafting to reduce demands can simultaneously reduce both exhaustion and performance (Demerouti et al., 2015). A few studies describe the dynamics between demands and resources as reciprocal, in which leadership plays a key role (Breevaart and Bakker, 2018); other studies found that demands and resources may operate through distinct processes that result in exhaustion or increased performance (Breevaart and Bakker, 2018; Schaufeli and Bakker, 2004). These findings make JD-R a valuable theory for managers, as it demonstrates how providing the right resources serves broader organizational goals.

In the context of neurodiversity, JD-R (Demerouti et al., 2001) is particularly relevant because it links aspects of work design to outcomes that may differ for neurodivergent employees. Previous research applied the theory to autistic employees and identified work characteristics that pose unique demands, including communication overload, ambiguous expectations, and efforts to maintain a good impression, which deplete energy over time (Tomczak and Kulikowski, 2024). Conversely, providing specific resources such as clear instructions, low-stimulus spaces, and flexible working hours can improve employee well-being (Petty et al., 2023). Another important factor in neurodiversity research is the decision to disclose, which, by itself, is stressful due to fear of stigma, social-psychological demands, and the fact that nondisclosure prevents access to important resources, thereby further increasing the risk of burnout (Tomczak and Kulikowski, 2024).

Specifically for individuals with ADHD, attentional patterns, executive functioning variability, and sensory sensitivity may shape how workplace demands and resources are experienced, appraised, and managed. From this perspective, the key issue is that while ADHD by itself does not constitute a job demand, it interacts with the environment in ways that can intensify or reduce experienced distress or lead to stronger engagement (Nagata et al., 2019). Accordingly, this study examines how adults with ADHD describe and manage the workplace demands they encounter, the resources that support sustainable functioning, and the broader conditions under which work becomes either depleting or sustainable.

Therefore, our research questions are as follows:

- RQ1: What workplace demands do individuals from an ADHD-related online discourse describe as shaping their work experience?
- RQ2: What workplace resources do ADHD-related online discourse describe as supporting sustainable effectiveness and positive outcomes?

## 2 Methods

### 2.1 Data collection

Reddit, an online platform for anonymous forums on various topics, has become an established source for qualitative health and organizational research because it captures naturally occurring discourse, peer support dynamics, and lived experience outside researcher-imposed structures (De Choudhury and De, 2014; Fiesler et al., 2024). Its large-scale participation, relative anonymity, and archived asynchronous discussions facilitate candid disclosure and preserve contextualized meaning-making over time (Andalibi et al., 2018). For stigmatized conditions such as ADHD, these online discussions may enhance data credibility by reducing social desirability bias and allowing participants to articulate workplace challenges and strengths in psychologically safer environments.

For the current study, we collected data from the Reddit forum (“subreddit”) r/ADHD, a large ADHD-focused subreddit, to capture heterogeneous experiences across occupations, employment arrangements, and career stages. As a diagnosis-based umbrella subreddit, r/ADHD allows users to self-describe their experiences rather than being pre-sorted into occupation-specific communities. To collect data on the experiences of people with ADHD in the workplace, we defined specific keywords. The threads retrieved through Reddit’s API contained at least one of the following keywords in their titles or descriptions: work, workplace, job, career, productivity, performance, schedule, deadlines, boss, manager, supervisor, coworkers, employee, employment, quit, burnout, stress, accommodations, flexibility, organize, and focus. After the initial screening, the research team manually filtered the data for relevance, retaining only threads containing information about workplace experiences. The research team applied additional filtering criteria to the data: they filtered out any threads with a score of less than 10 or fewer than 5 comments and considered only threads posted between 1/1/2023 and 12/31/2025. After the initial data sourcing and selection, a finalized list of 277 Reddit threads was generated. Researchers then systematically created several text documents containing information from the threads in the list. Each text file contained a single thread, including title, contents, creation date, URL, comments, and ID. Across all text files, there were 39,750 comments and a total of 1,226,113 words.

Several features of this dataset are methodologically important and shape the findings. First, the material reflects discourse produced within a large ADHD-centered online community rather than a neutral repository of individual workplace reports. As an ongoing peer support and discussion space, r/ADHD likely shapes both what is posted and how experiences are framed. Shared terms such as burnout, executive dysfunction, masking, accommodations, and rejection sensitivity may therefore function not only as individual descriptions but also as part of a collective meaning-making repertoire within the subreddit.

Second, because r/ADHD is an umbrella community rather than an occupation-specific forum, the dataset necessarily reflects substantial cross-role heterogeneity. Contributors likely represented a wide range of industries, job levels, work arrangements, and organizational contexts. As a result, the same workplace feature may have been experienced as a demand in one setting and as a resource in another. We therefore do not treat the dataset as representing a single coherent workplace context; rather, we interpret it as capturing cross-context patterns in how contributors’ descriptions of workplace strain and support.

Third, the use of engagement thresholds systematically shaped the dataset. By excluding threads with a score of less than 10 or fewer than 5 comments, the extraction produced a dataset of discussions that were more visible, resonant, or interaction-generating within the subreddit. These likely amplified narratives that attracted recognition, agreement, or elaboration from other users and may have reduced the presence of less visible, more routine, or less collectively endorsed experiences. Moreover, as with many health- and support-oriented online communities, the dataset may overrepresent visible, resonant, and distress-oriented narratives. Individuals may be more likely to post when work has become especially frustrating, conflict-laden, unsustainable, or emotionally salient than when work is routine or manageable. These patterns do not invalidate the data, but they do shape what type of experience is most likely to appear and circulate within the dataset.

Fourth, ADHD status in the dataset was self-reported. Contributors may have included formally diagnosed individuals, self-identified individuals, and those questioning or exploring whether ADHD described their experience. We therefore treat the dataset as ADHD-related workplace discourse produced by contributors who elected to participate in r/ADHD, which is appropriate for examining lived meaning-making but limits diagnostic precision. Together, these features informed our analytic stance from the outset. The findings are therefore interpreted as recurring patterns in perceived workplace experience and collective online meaning-making within r/ADHD, rather than as prevalence estimates or direct evidence of objective organizational structures.

In terms of ethical considerations, the study analyzed publicly accessible Reddit posts, in accordance with established internet research guidelines. The data were collected using an observational (passive) approach, meaning researchers gathered existing posts and comments from the r/ADHD subreddit. However, public accessibility was not treated as a sufficient ethical justification by itself. Because r/ADHD may function for many users as a support-oriented space in which workplace struggles, mental health concerns, and identity-related experiences are discussed, posts were treated as contextually sensitive rather than simply as public text.

To reduce the risk of traceability and preserve anonymity, identifying information such as usernames and specific job, workplace, or location details was removed or anonymized. No identifying information was retained in the reported findings, and identifying details were removed or anonymized during analysis and presentation. Excerpts were paraphrased where necessary to minimize searchability while preserving analytic meaning. No personal contact was made with any of the original posters. Consistent with the observational design, the study did not seek informed consent from individual users; however, interpretations were developed with attention to contextual integrity, participant vulnerability, and the limits of what could be ethically inferred from publicly accessible but socially sensitive discourse.

### 2.2 Data analysis

We conducted a reflexive thematic analysis (RTA) following Braun and Clarke’s six-phase approach (Braun and Clarke, 2021). The analysis was guided by a contextualist orientation, recognizing that Reddit posts reflect contributors’ interpretations of workplace experience while also being shaped by the discourse norms of the online community in which those experiences are discussed. Given the heterogeneity of occupations, work arrangements, and career stages represented in r/ADHD, our aim was not to infer a single, objective organizational structure but to identify demands, resources, and recurring cross-contextual patterns in how contributors perceived, described, and interpreted workplace conditions.

**Figure 1:**
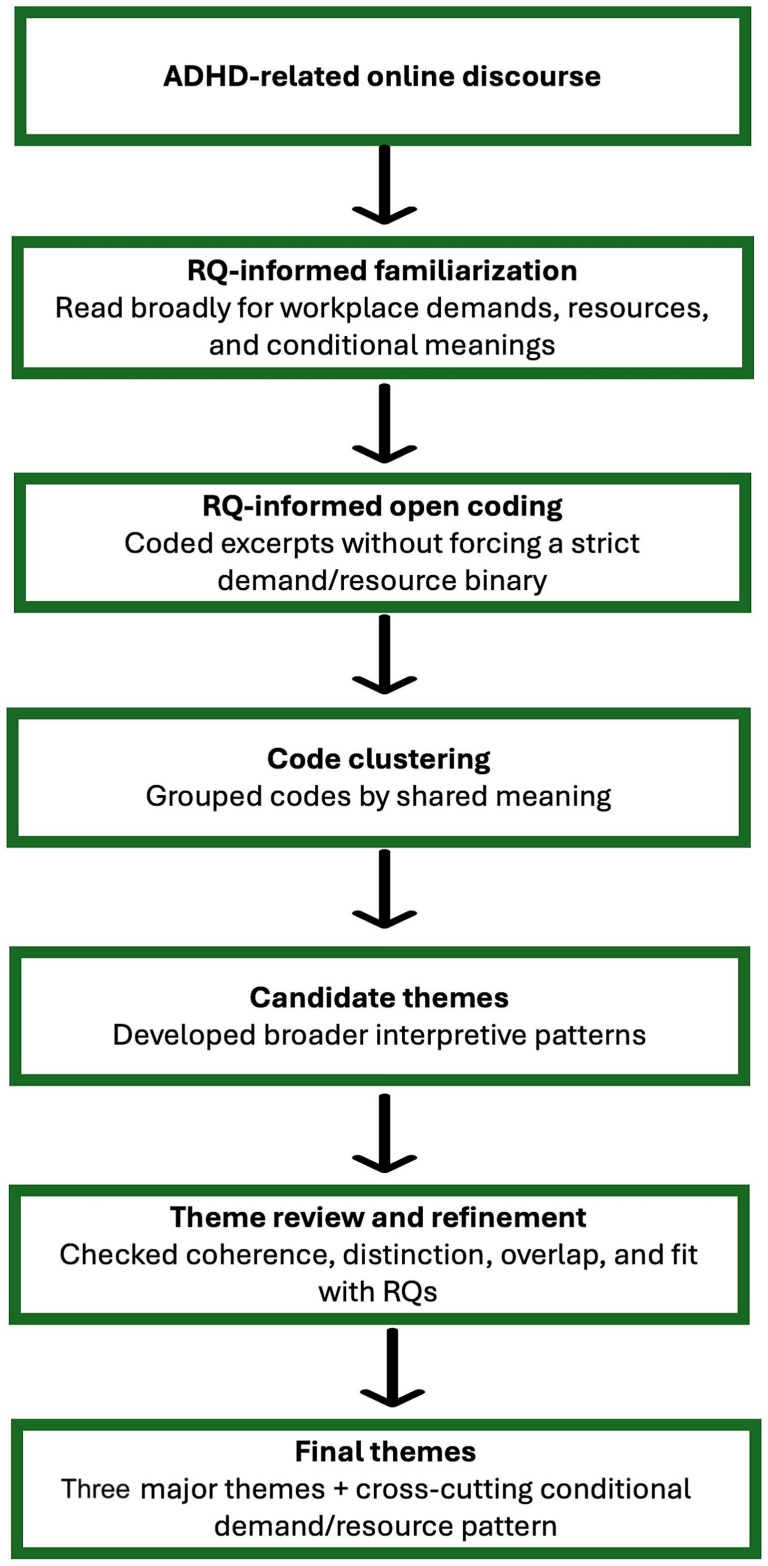
Analytic process and progression.

The analysis followed an abductive reflexive thematic analysis process. As shown in Figure 1, coding began with Authors 1, 2, and 4, who engaged closely with the ADHD-related online discourse and were guided by the study’s research questions concerning workplace demands and resources. It is important to mention researcher positionality. The research team approached the analysis with backgrounds in organizational and workplace research and with substantive interest in neurodiversity, work design, and sustainable employment. Author 1 provides theoretical, applied, and lived-experience perspectives within the neurodiversity paradigm, with significant global involvement in the neurodiversity community, offering a rich perspective on being neurodivergent. Author 2 contributes a theoretical background in organizational behavior and career management, whereas Author 4 draws on a background in social psychology and HR/labor relations. We recognized that these orientations may have increased our sensitivity to structural and relational explanations for strain, support, and sustainability. To address this, we used reflexive memoing and team discussion throughout the analytic process to examine how our assumptions shaped code development, theme boundaries, and theoretical interpretation. This process enabled the analysis to retain the inductive richness of participants’ accounts while interpreting theoretical aspects.

Initial coding was open and grounded in participant language, with attention to concrete workplace conditions, experiences, and meanings described in the data. Extracts were coded for how participants described work as difficult, supportive, or context-dependent, without forcing the data into a fixed demand/resource binary. After the initial coding, codes were clustered into broader patterns of shared meaning across the dataset and compared with the research questions. For example, referring to environments where employees were not sure about disclosing their condition, Author 1 identified *stigma, disclosure risk,* and *competence threat*, Author 2 proposed *disclosure agency, and double-edged sword,* and Author 4 identified *disclosure* and *privacy management*; Together, these formed the candidate theme “Disclosure turns diagnosis into a workplace credibility risk.” Author 3 joined to review these candidate themes, which were then reviewed and refined for coherence, distinctiveness, and then developed into themes. The Job Demands–Resources framework was used at this later interpretive stage as a sensitizing framework for understanding how the identified patterns related to perceived strain, support, and sustainability, without a fixed pre-existing coding template. Although some codes initially appeared to separate into workplace demands and workplace resources, this distinction was not always stable. Several workplace conditions, including disclosure, accommodations, flexibility, urgency, manager involvement, and structure, appeared to function as either demands or resources depending on how they were implemented and experienced. This focus on workplace conditions that could function as demands, resources, or both, depending on context, became our basis for the theme development - workplace features became harmful, supportive, or unsustainable.

Once six initial candidate themes were created by grouping codes with shared meaning, they were further refined by asking whether each theme captured a coherent pattern of meaning (Hoff and Strømme, 2025; Mok et al., 2026) was distinct from other themes, and advanced the analytic account of ADHD-related workplace experience. For example, “communication challenges” was not retained as a standalone theme because the coded extracts were not only about communication difficulties; they showed a broader pattern in which ADHD-related behaviors were interpreted as rudeness, defiance, laziness, or incompetence, resulting in differential treatment and exclusion. Similarly, “accommodations” was not retained as a standalone theme because accommodation-related extracts frequently overlapped with credibility risk, as well as with organizational design and fit. This process resulted in three final themes as described in the next section.

Table 1 describes the coding progression, from RQ-informed open coding of participant excerpts to codes and then to candidate themes. While initial codes remained close to participant language and observable workplace conditions, themes focused on broader patterns of shared meaning across the dataset.

**Table 1:** Coding-to-theme development.

Quotes	Codes	Candidate themes	Final theme
“Your boss is going to have a preconceived bias about ADHD.” “Revealing your diagnosis could impact your career development.” “Every mistake I made would be associated with my ADHD and I would be less trusted overall.” “I am often perceived as incompetent or lazy especially by my managers.” “Once you drop the D-word, people forget your competence and remember the stereotype.” “I disclosed and regret it. I feel like I’m now being ‘performance managed’…”	disclosure risk; stigma; anticipated bias; disclosure impact; career risk; promotion risk; disclosure consequences; loss of trust; manager misuse; termination risk; discrimination; credibility loss; diagnostic lens; performance scrutiny	Disclosure can turn diagnosis into a workplace credibility risk	Disclosure leads to differential treatment and status change
“My boss told me I’m insubordinate and argumentative.” “I ask a lot of question, not to challenge… but to understand.” “It 99% of the time always gets entirely misconstrued.” “Coworkers treating me different since they found out I have ADHD.” “My experience has been discriminatory — from casual misrepresentation… to diminishing my intelligence.” “This is working with adhd. Youre either too slow, too fast, doing the wrong thing, or pissing off coworkers.”	communication misinterpretation; manager judgment; relational threat; clarification needs; misunderstood intent; communication mismatch; misinterpretation; relational misunderstanding; intent/outcome gap; coworker stigma; changed treatment; disclosure aftermath; character judgment; perceived laziness; competence threat	ADHD-related differences lead to social exclusion	
“If you finish a project early you are rewarded with… more work.” “No matter how hard I work I still do not get to leave before 6.” “Sometimes we have to slow our pace and just do the bare minimum.” “You get no credit for showing up early or working thru lunch… but if you leave 10 min early it’s a big deal.” “As someone who’s lived with ADHD… the rigidity of this new system does not exactly set me up for success.”	competence punished; workload escalation; productivity expectations; time-based work norms; presenteeism; fixed schedule; visibility norms; punctuality pressure; unfair time expectations; rigid systems; time rules; poor environmental fit; sedentary work; monotony; under-stimulation; poor job fit	Neurotypical productivity norms are inconsistent with ADHD rhythms	Neurotypical workplace design and norms are not consistent with ADHD-specific needs
“I could not sit at a desk performing one task all day, every day.” “Working remotely has been a life changer.” “I can find the rhythm of when my brain focuses best, and follow it.” “I’m most successful and happy in jobs that are fast paced and physical.” “I need to have a career that lets me pursue various interests/projects.”	Poor environmental fit; sedentary work; monotony; under-stimulation; poor job fit; remote work; environmental flexibility; self-regulation; energy rhythm; strengths-based fit; stimulation; movement; job fit; variety; interest-based motivation; career fit	Workplace design determines whether work is leading to burnout	
“I cannot process verbal info during meetings.” “I miss meetings, though… alarms and pop-up reminders help.” “The hardest part of creating lists is the creating them part.” “Part of the problem… is chronic time blindness.” “Finding a job where people create documentation… helps ENORMOUSLY.” “Once we sat and talked, and set proper guidelines… my results went up.”	verbal processing; meeting demands; cognitive load; working memory; meeting recall; information retention; time blindness; prospective memory; compensatory tools; task initiation; planning load; organizing work; time estimation; deadline management; temporal regulation	Executive-function demands become invisible labor	Employees with ADHD are investing additional efforts that are not required of neurotypical employees
“I have a worksona that I maintain.” “I put a lot of energy into the workday, and there’s not much left for anything else.” “I’ve been doing my best to mask it and have been working for 10–12 + every day.” “Set boundaries when you can and work slower.” “I’ve been masking forever. it’s like my full time job is making other people comfortable.” “The reality is that I am overcompensating for being forgetful and the perfectionist and imposter syndrome in me has me doing way more than I should.” “Working hard and wowing managers does not actually make my life better or increase my pay… Burnout is not the participation trophy I am seeking.”	masking; concealment; burnout risk; professional persona; impression management; energy depletion; work-life imbalance; limited capacity; depletion; recovery deficit; sustainability strain; overcompensation; excessive hours; burnout; boundaries; pacing; recovery protection	Prolonged masking and overcompensation erode work sustainability	

## 3 Results

The reflexive thematic analysis generated three themes that describe how individuals in ADHD-related online discourse make sense of workplace demands, resources, and the conditions under which work becomes sustainable or unsustainable. Although the research questions distinguished between workplace demands and workplace resources, the analysis did not support a simple binary between the two. Theme development suggested that workplace conditions did not function uniformly as either demands or resources. Rather, the same condition could be experienced as supportive or burdensome depending on how it was implemented.

The themes that we identified are as follows: (1) Disclosure leads to differential treatment and status change; (2) Neurotypical workplace design and norms are not consistent with ADHD-specific needs; and (3) Employees with ADHD are investing additional efforts that are not required of neurotypical employees.

### 3.1 Theme 1: disclosure leads to differential treatment and status change

This theme captures how ADHD becomes conditional in workplace interactions once it was disclosed, suspected, or made visible through behavior, which can potentially undermine credibility and belonging. Across the discourse, participants described ADHD visibility as changing how managers and coworkers interpreted their competence, reliability, communication style, and professional standing.

In some accounts, ADHD visibility produced stigma, mistrust, and social distancing. Several participants framed disclosure as a risk to credibility and career standing. One participant warned that “*your boss is going to have a preconceived bias about ADHD*,” while another stated that “*revealing your diagnosis could impact your career development.*” These accounts suggest that disclosure was not understood simply as a personal choice or route to support, but as a decision that could alter how one’s future performance and professional potential were judged. This risk was particularly evident in the concern that “*every mistake I made would be associated with my ADHD and I would be less trusted overall*.” Here, ADHD becomes an interpretive lens through which ordinary mistakes are reclassified as evidence of unreliability or reduced competence.

The same pattern appeared when participants described ADHD-related behaviors being interpreted as character problems. For example, one participant reported, “*My boss told me I’m insubordinate and argumentative*,” while another explained, “*I ask a lot of question, not to challenge… but to understand*.” These extracts show a gap between intent and interpretation. Behaviors participants understood as clarification, processing, or attempts to perform well were sometimes read by others as defiance, difficulty, or poor attitude. Another participant summarized this relational strain by saying, “*It 99% of the time always gets entirely misconstrued*.” In these accounts, the workplace demand is not only managing ADHD-related behaviors but also managing how those behaviors are socially interpreted.

However, the theme also includes more supportive accounts of disclosure or ADHD visibility. One participant described a positive workplace context, stating, “*My team is very inclusive and I did not even think twice about telling them*.” Another described the effect of relational support: “*The support that I get from my manager and co-workers helps me to feel settled, reassured and completely appreciated*.” These accounts suggest that when teams are inclusive and psychologically safe, ADHD visibility can become a route to belonging rather than a source of stigma. In such contexts, disclosure may reduce the need for concealment and allow workplace relationships to become more supportive.

Taken together, this theme suggests that ADHD visibility produces a form of conditional workplace standing. Disclosure and ADHD-related differences were not experienced as inherently harmful or helpful, and their meaning depended on how the workplace reacts to them. When managers and coworkers respond through stigma or deficit assumptions, disclosure and ADHD-related behaviors can undermine credibility, increase scrutiny, and intensify social exclusion. When the workplace response is inclusive and supportive, the same visibility can enable understanding, reassurance, and belonging. The demand/resource overlap is therefore central to the theme: ADHD visibility becomes a demand when it exposes individuals to judgment, but a resource when it is met with trust, psychological safety, and practical support.

### 3.2 Theme 2: neurotypical workplace design and productivity norms are inconsistent with ADHD-specific needs

This theme captures how participants described workplace productivity systems as being organized around neurotypical assumptions about time, pace, visibility, and consistency. In these accounts, “good work” was often measured through fixed schedules, punctuality, time-at-desk, visible busyness, and steady output rather than through actual task completion or sustainable performance. People with ADHD may have different needs for regulation, stimulation, flexibility, movement, and recovery, as well as work rhythms, that can be more variable, sprint-like, energy-dependent, or responsive to urgency. Therefore, alignment (or misalignment) between work design and these needs and rhythms impacts productivity.

Several extracts illustrated how productivity was tied to time and visibility rather than outcomes. One participant described the frustration of finishing work efficiently but being “*rewarded with… more work*,” suggesting that competence could become a pathway to workload escalation rather than recognition or recovery. Another participant explained, “*No matter how hard I work I still do not get to leave before 6*,” highlighting how fixed schedules can override actual productivity. A similar frustration appeared in the account that “*you get no credit for showing up early or working thru lunch… but if you leave 10 min early it’s a big deal*.” Together, these accounts suggest that workplace norms often prioritize visible compliance with time expectations over the quality, pace, or completion of work. Moreover, rigid systems that leave little room for ADHD-related regulation created additional strain. One participant stated, “*As someone who’s lived with ADHD… the rigidity of this new system does not exactly set me up for success*.” This points to a broader pattern in which workplace systems were experienced as disabling not simply because work was difficult, as the demand is not only task completion but the need to perform in a way that matches organizational expectations of consistency, punctuality, and visible presence.

Participants also referred to their needs for sufficient stimulation, movement, and variation. One participant described needing “*a career that lets me pursue various interests/projects*,” while another explained, “*I could not sit at a desk performing one task all day, every day*.” These accounts point to the same underlying pattern: work that is narrow, sedentary, or repetitive can be difficult to sustain, while work that allows variety, movement, and interest-based engagement is more likely to support ADHD-related regulation. This was also reflected in the statement, “*I’m most successful and happy in jobs that are fast paced and physical*.” Thus, pace and movement that can be seen distractions from productivity are actually conditions that make productivity and satisfaction more possible.

The pattern suggests that participants often experienced a mismatch between ADHD work rhythms and conventional productivity models. Some accounts described productivity as nonlinear: participants could work intensely, finish quickly, or perform effectively under the right conditions, yet still be constrained by fixed hours, visibility norms, and expectations of constant output. This creates a paradox in which employees may be penalized both for struggling with rigid time expectations and for being efficient within them. Efficiency does not necessarily produce autonomy or rest; instead, it may produce more work, higher expectations, or resentment.

At the same time, some participants described flexibility as a resource when it allowed them to regulate attention and energy more effectively. One participant stated, “*Working remotely has been a life changer*,” while another explained, “I *can find the rhythm of when my brain focuses best, and follow it.*” These accounts suggest that flexible work was not simply valued for convenience: allowing participants to self-regulate (e.g., organize work around periods of focus, manage environmental demands, and work in ways that are better aligned with their fluctuating capacity) supported productivity better than visible, linear, time-bound performance norms.

### 3.3 Theme 3: employees with ADHD are investing additional efforts that are not required of neurotypical employees

This theme captures how participants described ordinary workplace tasks as requiring substantial additional labor that was often invisible to others. Because of difficulties with executive functioning that are often characteristic to people with ADHD, beyond completing assigned tasks their additional demands regarding processing verbal information, remembering meetings, tracking time, initiating tasks, organizing work, estimating deadlines, and translating expectations into action. Participants’ accounts suggested that these forms of cognitive labor became especially difficult when workplaces assumed that employees could internally manage memory, time, attention, and prioritization without explicit supportive measures.

Several extracts illustrate how routine workplace practices created hidden cognitive demands. One participant explained, “*I cannot process verbal info during meetings*,” while another described how they had “*so much trouble remembering what was discussed in meetings*.” These accounts show that meetings were not experienced only as social or informational events; they also required rapid auditory processing, working memory, note-taking, and later recall. Similarly, the statement “*I miss meetings, though… alarms and pop-up reminders help*” points to prospective memory as an ongoing workplace demand. The problem was not simply forgetting, but needing to maintain systems that made time, commitments, and transitions visible. Another participant described task organization itself as labor, stating, “*The hardest part of creating lists is the creating them part*,” while another identified “*chronic time blindness*” as part of the problem. Together, these extracts show that planning, estimating, remembering, and initiating were experienced as work before the visible work could even begin.

However, the same accounts also showed that this labor became more manageable when it was externalized through documentation, reminders, clear expectations, and collaborative structure, and when others recognized these supports as legitimate rather than as signs of incompetence. Participants described external supports as highly effective when they reduced reliance on internal memory or self-generated structure. One participant stated that “*finding a job where people create documentation… helps ENORMOUSLY*,” suggesting that written records, task outlines, and shared documentation can make work more accessible by externalizing information that might otherwise have to be held in memory. Another participant described improvement after collaborative clarification: “*Once we sat and talked, and set proper guidelines… my results went up*.” This extract is particularly important because it shows that executive-function support was not only about individual coping tools; it also involved workplace recognition, shared expectations, and manager or team participation.

Beyond the executive functioning labor, in less supportive contexts participants engage in masking and overcompensation, which add even more strain. One participant stated, “*I have a worksona that I maintain*,” suggesting that work required an ongoing performance of acceptability, professionalism, or neurotypical competence. Others described the energetic cost of this performance: “*I put a lot of energy into the workday, and there’s not much left for anything else*,” and “*I’ve been doing my best to mask it and have been working for 10–12 + every day*.” These extracts suggest that participants could sometimes meet workplace expectations, but only by drawing heavily on personal reserves. The issue, then, was not always immediate inability to perform, but the unsustainable conditions under which performance was achieved. Yet, some participants were able to protect themselves through bounded effort, e.g., setting limits, pacing and protecting recovery time. The advice to “*set boundaries when you can and work slower*” refers to ensuring that work expectations are contained enough to be sustainable: workload is realistic, recovery is possible, and performance does not depend on constant overextension. Moreover, when participants did not have to suppress their needs, perform a professional persona, or compensate for poor fit through excessive effort, work became more manageable.

The pattern across these accounts suggests that executive-function difficulties became most demanding when they were treated as individual failures rather than systemic work misalignment issues. While participants described breakdowns in memory, time estimation, task initiation, and meeting processing, as well as the emotional labor of masking and the need to overcompensate for difficulties, they also identified practical measures that helped: alarms, reminders, written documentation, clear guidelines, and collaborative structure. In this sense, executive-function labor became manageable when it was acknowledged and addressed by moving out of the individual’s head and into the work environment.

### 3.4 Cross-cutting pattern: demands and resources are conditional rather than fixed

While all three themes described above describe potential pathways to burnout (social treatment, work misalignment, and additional effort), interestingly, across the themes the analysis suggests that this is not always the case. Workplace conditions were not inherently demands or resources; instead, their function depended on whether they were experienced as fitting their needs, reliable, clear, and sustainable. Disclosure, accommodations, structure, flexibility, deadlines, manager involvement, and even masking could become supportive or harmful depending on the context. For example, one contributor posted, “*I work remotely now so I can log in exactly on time, but when I commuted I struggled with lateness because commuting requires you to do so many steps*”, demonstrating flexible work as a resource. By contrast, another contributor wrote, “*Without external accountability, I find myself getting distracted more often,*” indicating that being in-person may help with sustainable work. This suggests that a simple demand/resource binary may not fully capture ADHD-related workplace experience, as the same condition can support effectiveness in one context and undermine sustainability in another: just as some contributors mentioned that flexibility can help when it allows regulation, others noted that it can be overwhelming when it removes structure. Similarly, disclosure was mentioned as potentially unlocking support but also exposing people to stigma; time urgency can mobilize performance for some people (*“Deadlines help me focus.”*), however chronic urgency can produce burnout and ineffective planning (*“I can only get things done when it’s an emergency.”*); structure can clarify work, but also become controlling in case of rigidity.

This notion explains why participants sometimes described the same workplace feature in contradictory ways: ADHD-related workplace experience is shaped less by whether a feature is formally a demand or a resource, and more how it is implemented. Therefore, while our initial questions focused on demands and resources, our findings show that the demand/resource distinction is dynamic, as the as the interpretation of different workplace conditions can shift in meaning.

## 4 Discussion

Altogether, our findings are best understood as a JD–R-informed interpretive account of how workplace strain and support are described within ADHD-related online discourse, rather than as direct representations of organizational structures or mechanisms. The three interpretive themes we identified capture recurring patterns in how contributors make sense of their work experiences, particularly in contexts where identity, attentional regulation, and organizational expectations intersect. We recognize that these themes represent multiple well-established organizational dynamics as they co-occur in participant accounts. Across themes, contributors did not describe demands and resources as isolated job characteristics; rather, they framed them as interconnected features of perceived work environments that shape whether work becomes sustainable or depleting, and this identified interconnection is our main contribution to theory.

One theme describes how disclosure can threaten workplace credibility and competence. These accounts resonate with constructs such as psychological safety (Edmondson, 2018) and leader–member exchange (Graen and Uhl-Bien, 1995), as participants may sometimes be required to keep defending their status. Moreover, we also identified how neurodivergent identity can impact workplace belonging, which aligns with research on concealable stigma and identity management (Clair et al., 2005). The relational aspects can impose additional demands or serve as a source of support, but emphasize the ongoing regulatory effort associated with anticipating, managing, or avoiding stigma. In this theme, identity is not treated as a static attribute but as a context-dependent condition.

Another theme refers to the alignment, or misalignment, between workplace structures and their ADHD-related regulation needs. These accounts are consistent with established research on person–environment and person–job fit (see Caldwell et al., 2003; Edwards, 2008; Zeng and Hu, 2024), but extend this perspective by emphasizing dynamic, moment-to-moment regulation rather than relatively stable compatibility. Contributors’ narratives suggest that strain is not attributed solely to workload intensity, but to whether work structures allow for adaptive regulation of attention and energy over time.

The third theme described additional executive-function demands that are posed by having ADHD-related needs, and that are taken for granted by neurotypical colleagues. While prior research identified challenges that people with ADHD may face in the workplace (Liebel et al., 2023; McDowall et al., 2025), not all supervisors and coworkers may be aware of them (Ali et al., 2024) which can result in additional strain. At the same time, the value-in-diversity perspective (Cox and Blake, 1991) claims that a diverse organization, which can refer to diversity in cognitive functioning (Doyle and McDowall, 2022), can enjoy improved outcomes, if this diversity is properly managed and supported. While people with ADHD may experience difficulties with executive functioning, they may also have strengths, and/or develop strategies that will become sources of strength (Crook and McDowall, 2024) particularly if supported by the organization.

Taken together, these patterns suggest that contributors may not experience workplace demands and resources as discrete inputs, i.e., characteristics of tasks or roles, but as conditions, emergent properties of broader work arrangements. Importantly, these findings should be interpreted within the context of the data source. The patterns identified here reflect collective meaning-making within an online ADHD community, shaped by shared language, engagement dynamics, and the likelihood that more salient or distress-oriented experiences are more frequently discussed. As such, the themes do not represent objective features of workplaces, but rather recurring interpretive patterns in how contributors describe and make sense of their experiences. This distinction is critical for maintaining alignment between the nature of the data and the scope of the claims.

From this perspective, ADHD is best understood as an individual-level source of regulatory variability that shapes how workplace conditions are experienced, appraised, and managed. Contributors’ accounts consistently frame strain not as inherent to ADHD itself, but as arising where workplace structures are misaligned with individual needs, and conversely, alignment can be interpreted as a resource. This interpretation aligns with neurodiversity scholarship that emphasizes the role of environmental design and contextual support in shaping outcomes (Ali et al., 2024).

Overall, the contribution of this study lies in providing a contextualized, discourse-informed interpretation of JD–R processes within ADHD-related workplace narratives. Rather than proposing fully validated theoretical constructs or organizational prescriptions, the findings offer provisional interpretive themes that may guide future empirical research. These findings should therefore be treated as hypothesis-generating rather than as a basis for organizational design conclusions.

### 4.1 Practical implications

Although the present study does not support direct organizational prescriptions, the findings identify several practice-facing areas that warrant reflection and further examination. First, given the importance of disclosure and social acceptance as described by the contributors, it is important to invest effort in workplace norms that make disclosure feel safe, useful, and non-punitive. It is also important to ensure that evaluation systems are clear, consistent, and transparent, such that employees can demonstrate their competence. Moreover, we call for sensitivity to interpersonal aspects such as communication difficulties, and encouraging inclusion, in terms of uniqueness and belonging. Next, it is recommended to improve the fit between work design and employee needs: for some employees it may be flexibility, for other a combination of autonomy and control, however the ability to adapt workplace conditions will be beneficial not just for employees with ADHD but for everyone. We also recommend increasing awareness of ADHD-related needs, which will make executive-functioning demands visible and thus ensure the demands are reasonable and overwhelming. Finally, awareness of the additional labor should also help with sustained performance: while short-term increased effort may be acceptable, managers need to ensure that stress does not become permanent and to allow recovery.

To sum up, we suggest a broader approach to workplace support for employees with ADHD, beyond isolated accommodations. Moreover, while our study focused on people with ADHD, these recommendations may benefit the organization, by becoming more inclusive. With that, this study provides a contextualized, discourse-informed interpretation of JD–R processes within ADHD-related workplace narratives. Therefore, our findings reflect collective meaning-making rather than objective features of workplaces, and its implications should be treated as cautious, hypothesis-generating considerations. This boundary is critical for ensuring that our insights are used as considerations for reflection and examination, rather than as direct prescriptions for organizational change.

### 4.2 Limitations

Several limitations warrant consideration when interpreting this study’s findings. First, the data are self-selected and publicly available, which limits our ability to characterize the broader population of individuals with or who identify with ADHD. Moreover, ADHD status is self-reported and may include formally diagnosed, self-identified, or questioning individuals. While this limits clinical verification, identity-based self-location is central to understanding lived experience within neurodiversity discourse. Reddit also lacks systematic demographic data, limiting the specificity of demographic analysis and the generalizability of statistical inferences. We do not know the total number of individuals represented in the dataset, their demographic characteristics, occupational contexts, or the extent to which these experiences generalize beyond Reddit users.

Additionally, online communities may overrepresent individuals experiencing distress, potentially amplifying demand-related narratives and introducing variability in who is represented in the sample and which experiences are most likely to be shared. To enhance dependability and confirmability, the analytic procedures emphasized transparency, systematic coding, and reflexive engagement with the researchers’ assumptions. Findings are therefore intended to advance JD–R-informed theoretical understanding of perceived workplace demands and resources rather than to estimate population prevalence.

Next, the data are fragmented and inherently constrained by the structure of online posts and comments. Each thread provides partial insight into experiences and perspectives, often lacking context or longitudinal detail, making it difficult to capture the full complexity of participants’ work lives. The observational nature of the data collection impedes clarification or follow-up. Researchers were unable to ask questions, probe ambiguous statements, or verify the accuracy of self-reported experiences. Consequently, interpretations rely solely on what participants chose to disclose, thereby omitting nuanced or sensitive details.

### 4.3 Future research

Future research should expand across platforms and subcommunities, integrate mixed-methods approaches, and incorporate longitudinal designs to differentiate perceived structural patterns from episodic distress. Experimental or quasi-experimental studies are needed to test whether changes in work design conditions, such as structured milestones, transparent evaluation systems, and predictable feedback practices, influence outcomes for neurodivergent workers. Research should also examine disclosure timing, managerial response, and organizational culture as moderators of workplace experience.

## Data Availability

Publicly available datasets were analyzed in this study. This data can be found at: www.Reddit.com/r/ADHD.
